# Supplementary material for: Intravenous Oncolytic Vaccinia Virus Therapy Results in a Differential Immune Response between Cancer Patients
Source: Cancers (Basel). 2022 Apr 27;14(9):2181. doi: 10.3390/cancers14092181 (PMC9103071; doi:10.3390/cancers14092181)
Supplement: Supplementary file 1 [file cancers-14-02181-s001.zip › cancers-1650781-supplementary.pdf]

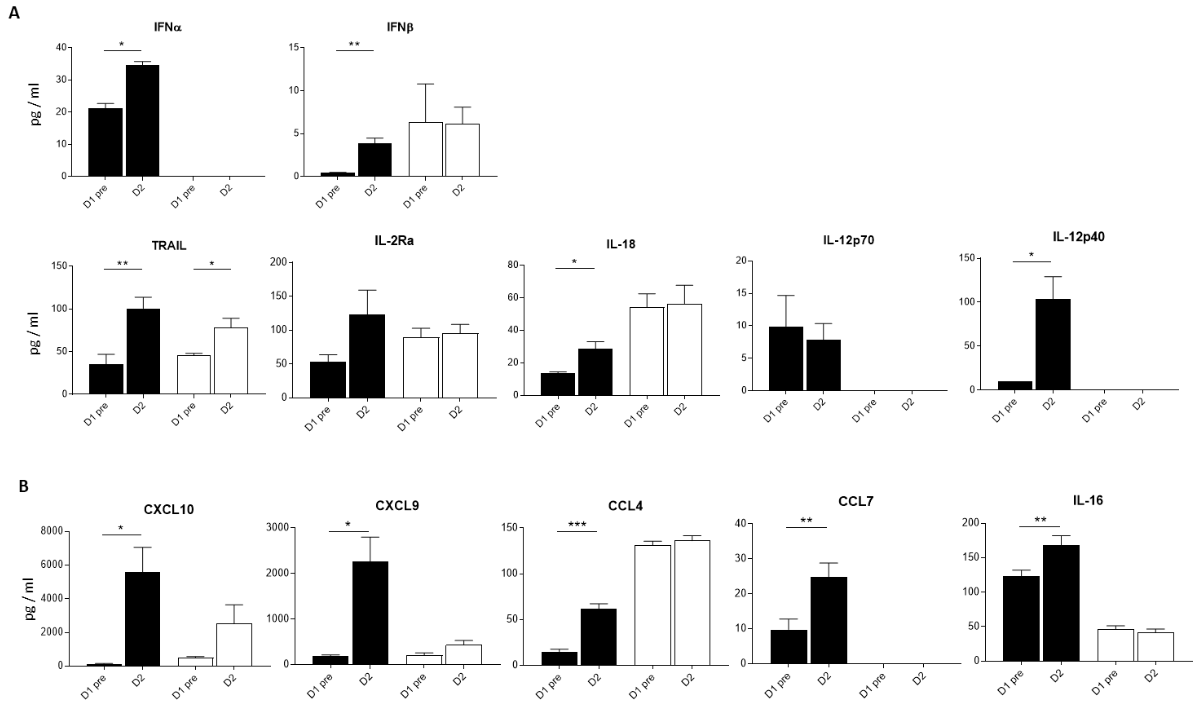

Supplementary Figure S1. Differential IFN and inflammatory cytokine **(A)** and chemokine (**B**) response to *Pexa-Vec* in Responder (black; n = 4) and Exhausted (white; n = 5) patients. Data is shown as baseline compared to D2 plasma concentrations (pg/ml); * $p < 0.05$, ** $p < 0.01$, *** $p < 0.001$.

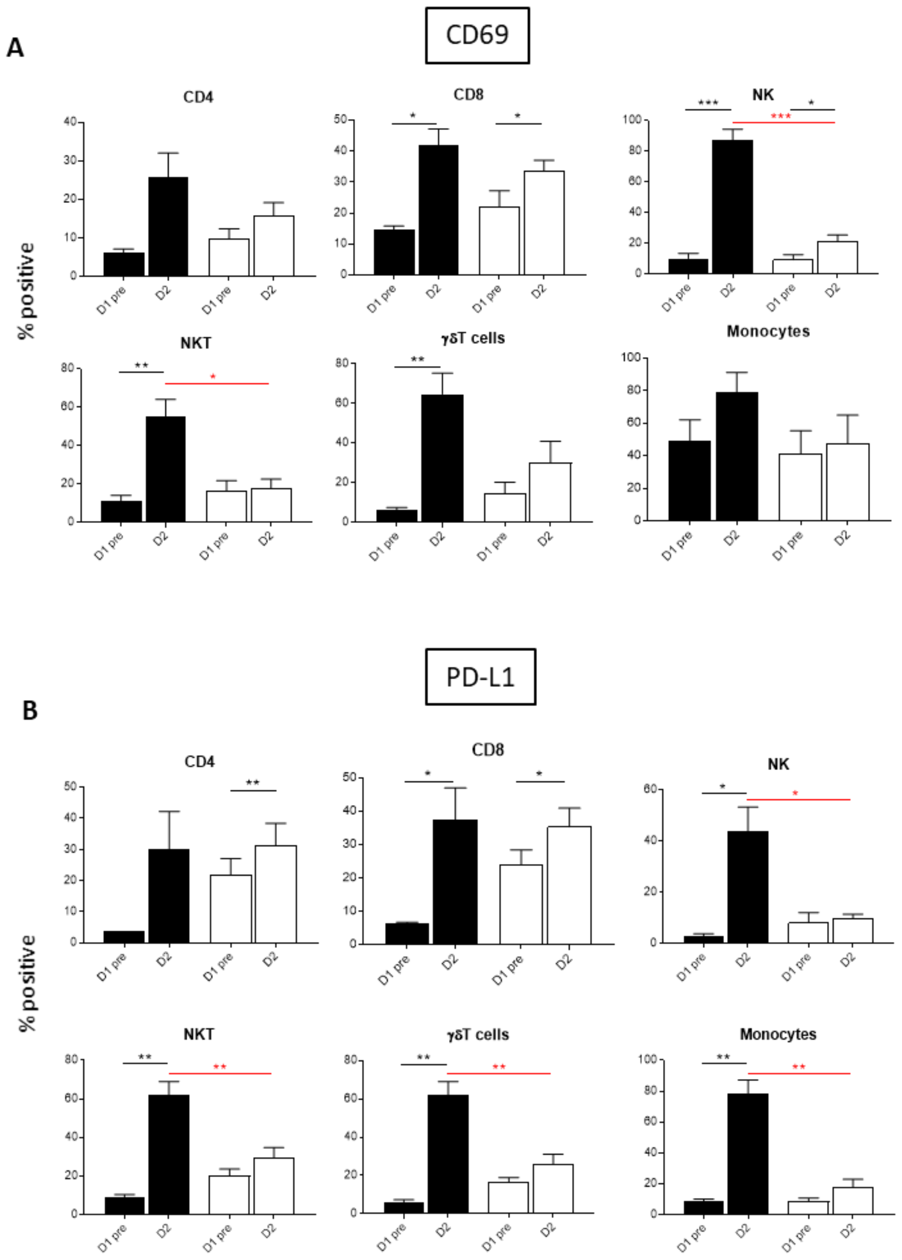

Supplementary Figure S2. Differential activation of immune cell populations following *Pexa-Vec* infusion. Differential expression of **(A)** CD69 in immune cell populations and **(B)** PD-L1 expression in immune cell populations in Responder (black; n = 4) and Exhausted (white; n = 4/5) patients. Data is shown as baseline compared to D2 within each patient group or Responder compared to Exhausted at D2 (statistical significance shown in black or red, respectively) for positive expression; * $p < 0.05$, ** $p < 0.01$, *** $p < 0.001$.
